# Introducing a Pole Concept for Nodule Growth in the Thyroid Gland: Taller-than-Wide Shape, Frequency, Location and Risk of Malignancy of Thyroid Nodules in an Area with Iodine Deficiency

**DOI:** 10.3390/jcm11092549

**Published:** 2022-05-01

**Authors:** Manuela Petersen, Simone A. Schenke, Michael Zimny, Rainer Görges, Michael Grunert, Daniel Groener, Philipp Seifert, Peter E. Stömmer, Michael C. Kreissl, Alexander R. Stahl, on behalf of the German TIRADS Study Group

**Affiliations:** 1 Department of General, Visceral, Vascular and Transplant Surgery, University Hospital Magdeburg, Leipziger Straße 44, 39120 Magdeburg, Germany; 2 Department and Institute of Nuclear Medicine, Hospital Bayreuth, 95445 Bayreuth, Germany; simoneschenke@web.de; 3 Division of Nuclear Medicine, Department of Radiology and Nuclear Medicine, University Hospital Magdeburg, 39120 Magdeburg, Germany; michael.kreissl@med.ovgu.de; 4 Institute for Nuclear Medicine Hanau, 63450 Hanau, Germany; zimny@nuklearmedizin-hanau.de; 5 Clinic for Nuclear Medicine, University Hospital Essen, 45147 Essen, Germany; rainer.goerges@uni-due.de; 6 Department of Nuclear Medicine, German Armed Forces Hospital Ulm, 89081 Ulm, Germany; michael.grunert@uni-ulm.de; 7 Department of Nuclear Medicine, University Hospital Frankfurt, 60590 Frankfurt, Germany; daniel.groener@gmx.de; 8 Clinic of Nuclear Medicine, University Hospital Jena, 07749 Jena, Germany; philipp.seifert@med.uni-jena.de; 9 Institute for Pathology, Pathologicum, 86150 Augsburg, Germany; profstoemmer@gmail.com; 10 Research Campus STIMULATE, Otto-von-Guericke University, 39106 Magdeburg, Germany; 11 Institute for Radiology and Nuclear Medicine, RIZ, 86150 Augsburg, Germany; dr.alexander.stahl@gmx.de

**Keywords:** TIRADS, taller-than-wide, TTW nodules, risk of malignancy, Zuckerkandl’s tubercle, posterior horn, posteroinferior horn, pole concept of goiter growth

## Abstract

**Purpose**: (i) To examine the criterion taller-than-wide (TTW) for the sonographic assessment of thyroid nodules in areas of iodine deficiency in terms of frequency, anatomical distribution within the thyroid gland and risk of malignancy. (ii) To develop a model for nodule growth in the thyroid gland. **Methods:** German multicenter study consisting of two parts. In the prospective part, thyroid nodules were sonographically measured in all three dimensions, location within the thyroid gland and contact to a protrusion-like formation (horn) in the dorsal position of thyroid gland was noted. In addition, further sonographic features such as the composition, echogenity, margins and calcifications were investigated. All nodules from the prospective part were assessed for malignancy as part of clinical routine at the decision of the treating physician adhering to institutionally based algorithms. In the retrospective part, only nodules with fine needle aspiration and/or histology were included. The risk of malignancy in TTW nodules was determined by correlating them with cyotological and histological results. **Results:** Prospective part: out of 441 consecutively evaluated thyroid nodules, 6 were found to be malignant (1.4%, 95% CI 0.6–2.7%). Among the 74 TTW nodules (17%), 1 was malignant (1%, 95% CI 0–4%). TTW nodules were more often located in the dorsal half of the thyroid than non-TTW nodules (factor 2.3, *p* = 0.01, 95% CI 2.1–2.5) and more often located in close proximity to a horn than non-TTW nodules (factor 3.0, *p* = 0.01, 95% CI 2.4–3.8). Retrospective part: out of 1315 histologically and/or cytologically confirmed thyroid nodules, 163 TTW nodules were retrieved and retrospectively analyzed. A TTW nodule was 1.7 times more often benign when it was dorsal (95% CI 1.1–2.5) and 2.5 times more often benign when it was associated with a horn (95% CI 1.2–5.3). The overall probability of malignancy for TTW nodules was 38% (95% CI 30–46%) in this highly preselected patient group. Conclusion: TTW nodules are common in iodine deficient areas. They are often located in the dorsal half of the thyroid gland and are frequently associated with a dorsal protrusion-like formation (horn) of the thyroid. Obviously, the shape of benign nodules follows distinct anatomical preconditions within the thyroid gland. The frequency of TTW nodules and their predominant benignity can be explained by a pole concept of goiter growth. The difference between the low malignancy risk of TTW nodules found on a prospective basis and the high risk found retrospectively may be the result of a positive preselection in the latter.

## 1. Introduction

Iodine deficiency is an important risk factor in the development of nodular thyroid disease [1]. More than 30% of the German population suffer from mild to moderate iodine deficiency. Although substantial progress has been made in recent decades in eliminating iodine deficiency, functional thyroid disorders and goiter are still prevalent [2]. The prevalence of thyroid nodules ranges from 12.5% in young men to over 80% in older woman [3]. The clinical challenge is to reliably detect malignant nodules while avoiding unnecessary interventions for benign lesions [4].

High-resolution ultrasound (US) is the most important imaging modality for the characterization of thyroid nodules. Different research groups developed US-based tools for stratifying the risk of malignancy of thyroid nodules using a combination of suspicious ultrasound features. In 2009, the terminology of the “Thyroid Imaging Reporting and Data System” (TIRADS) was introduced [5], based on the “Breast Imaging Reporting and Data System” (BIRADS), which has been established for breast tumors for many years. Whereas some US criteria for malignancy in thyroid nodules were already known to the community—such as hypoechogenicity and irregular margin—others were newly introduced, such as a taller-than-wide (TTW) shape [5,6,7,8,9,10,11].

According to the definition, TTW thyroid nodules have a larger sagittal (depth) than transverse (width) diameter. The longitudinal diameter (length) along the axis of the thyroid lobe is not included into the definition. A TTW shape as seen on US is associated with malignancy according to a variety of studies. Remonti et al., reported a sensitivity of 26.7% and a specificity of 96.6% [12]. In two meta-analyses, TTW shape was the single US feature with the highest odds ratio for malignancy [13,14]. The TTW shape of nodules was an independent predictor of malignancy (sensitivity: 15.2–53.0%; specificity: 88.2–98.7%; PPV: 47.3–77.5%) [7,9,15,16,17,18]. The malignancy risk of TTW nodules was further modified by their composition and echogenicity [9,15]. It was higher in solid or hypoechoic nodules (77.0–87.7%) as compared to partially cystic or iso-/hyperechoic nodules (10.5–31.3%) [9,15,19].

Although not synonymous, TTW shape is also referred to as non-parallel orientation. This term indicates the concept of TTW growth as a sign for malignancy. It implicates that, malignancies within the thyroid gland tend to grow against the given orientation of the thyroid gland axis, which is along the long axis of the thyroid gland. In contrast, benign nodules appear to respect this orientation by growing parallel with respect to the long axis [20].

In our study we extended this concept of parallelism to include a known protrusion of the thyroid gland towards the back named Zuckerkandl’s tubercle. Throughout this article, Zuckerkandl’s tubercle is referred to as posterior horn as was done by its first descriptions [21,22]. Along with the development of goiter, the posterior horn is known to be involved in overall thyroid growth and to harbor thyroid nodules [23]. Our aim was to investigate if this protrusion—which by its nature is TTW—alters the growth pattern of benign nodules in this region, such that benign nodules assume a TTW shape. A TTW shape of benign nodules, however, at such location would still be parallel with regard to the protrusion.

On US, the posterior horn is hard to see as it is often hidden behind the trachea [24,25]. On the other hand, particularly in goitrous growth, a dorsocaudal protrusion at each thyroid gland may be seen. This extension has no proper name in literature. It is indirectly named as “cleft sign” in autoimmune thyroid disease, meaning the fibrous duplicature of the thyroid capsule developing between this dorsocaudal extension and the back surface of the lower thyroid pole [26]. It is unclear to the authors, if such dorsocaudal protrusion may be considered as a variant of the posterior horn. To make a distinction, this dorsocaudal protrusion is herewith introduced as posteroinferior horn.

Throughout this paper, any extremity of the thyroid gland, i.e., the upper and lower end of each thyroid lobe, the thyroid isthmus and any form of horn, is referred to as a pole. Such poles are the basis for a concept for nodule growth to be developed in the Section 4 based on the results of the study. As a hypothesis, we assume the configuration of thyroid poles to channel the shape of nodules. In this context, in an area of iodine deficiency, we investigated the following questions regarding TTW configuration:

1. How frequent is TTW growth of thyroid nodules?
2. Where are TTW nodules located within the thyroid gland?
3. What is the risk for malignancy in TTW nodules?
4. Finally, a pole concept for nodule growth is developed.

## 2. Materials and Methods

The multicentric data collection was conducted according to the guidelines of the Declaration of Helsinki and approved by the Ethics Committee of the Medical Faculty of the University Hospital of Duisburg-Essen, Germany (protocol code: 16-7022-BO, 04-AUG-2016, date of approval at 4 August 2016).

This study is divided into two parts. The first part is prospective in nature; 441 consecutive thyroid nodules with a minimum diameter of ≥7 mm in each direction were examined for TTW status. Only initial presentations (first US examinations) were considered. The patients were recruited from six centers distributed across Germany, which all belong to the “German TIRADS Study Group” (GTSG, www.tirads.de, accessed on 2 April 2022). Experienced examiners measured the nodules in all three spatial dimensions using US.

**Figure 1:**
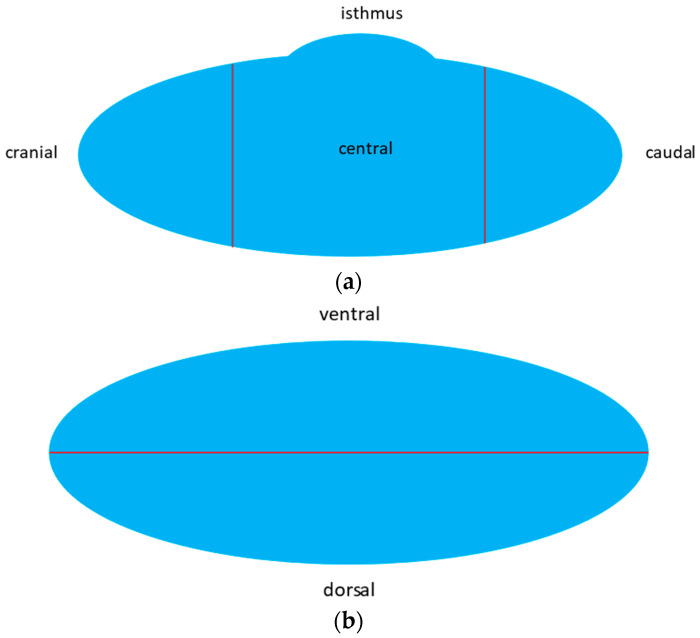

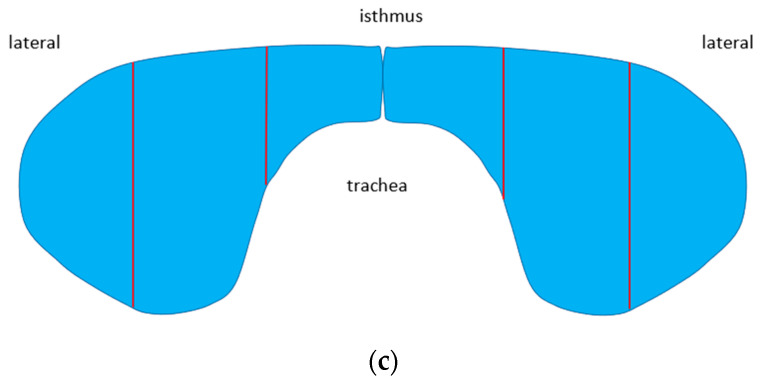
(**a**) Dimension craniocaudally: cranial–central–caudal. (**b**) Dimension ventrodorsally: ventral–dorsal. (**c**) Dimension horizontally: lateral–medial (isthmus).

The location of each TTW nodule within the thyroid gland was described in three dimensions (Figure 1a–c).

Using a uniform Excel file, investigators were instructed to assign each nodule to a particular location according to the center of the nodule in the respective dimension. The position in which the largest part of the nodule was located was decisive for specifying the nodule location. In cases of doubt, double assignment in each dimension was allowed, e.g., cranio-central. When a nodule was assigned to the thyroid isthmus, no further assignments in the craniocaudal and ventrodorsal dimension were recorded.

**Figure 2:**
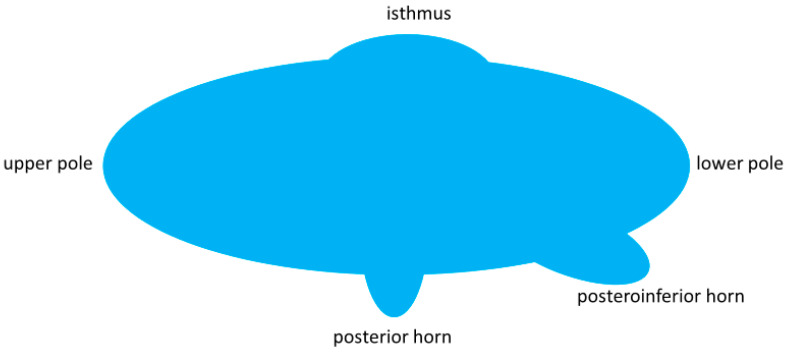
Stylized sagittal view of the thyroid gland. Note the posterior and the posteroinferior horn as protrusions at the back. These protrusions as well as the upper and lower extremities of the thyroid lobe and the isthmus are introduced as poles of the thyroid gland for the purpose of a pole concept of nodule growth to be developed in the Section 4.

**Figure 3:**
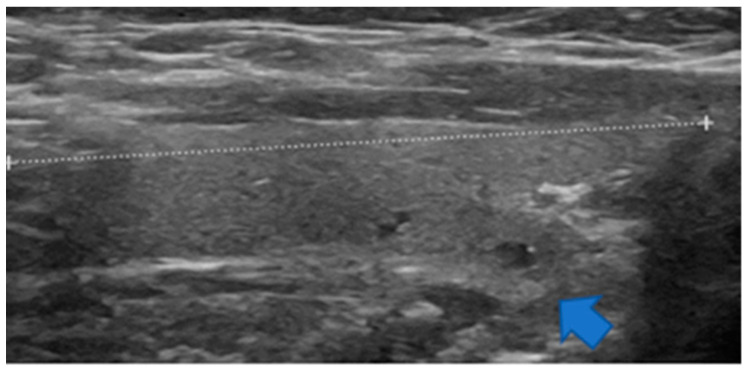
Sagittal view of a non-enlarged thyroid lobe. At the lower portion, there is a downward and backward protrusion named the posteroinferior horn herein (blue arrow).

In addition, it was noted if the thyroid nodule had contact to a horn at the posterior margin of the thyroid gland. We assumed a horn if the back surface line of a thyroid lobe appeared to be interrupted by an antiparallel protrusion of the thyroid tissue backwards or backwards and downwards. The backward protrusion was named a posterior horn according to literature. In analogy, a protrusion backward and downward was named a posteroinferior horn (Figure 2 and Figure 3). For further analysis and to simplify, both forms were often summarized as horn. We defined a nodule to have contact to a horn, if (i) such protrusion was recognizable on the images and (ii) the nodule extended into such horn, i.e., the dorsal contour of the nodule protruded from the back part of the thyroid gland. 

The second part of the study was retrospective. It was conducted in order to assess the risk of malignancy in TTW nodules depending on their location within the thyroid gland. In order to achieve this aim, TTW nodules were retrieved from databases of six cooperating diagnostic and therapeutic thyroid centers (GTSG). These databases are continuously maintained (updated) and contain US image data as well as information on the histological or cytological diagnosis and have been previously used for the evaluation of TIRADS criteria in Germany [3,27,28]. The databases are preselected in that only nodules with cytological and/or histological confirmation were included and autonomously functioning thyroid nodules were excluded. The data already assessed (among which width, depth and length of each nodule in mm) were re-used and the according images stored in the image archival system (PACS, picture archiving and computing system) were re-evaluated regarding the location of nodules within the thyroid gland.

Nodules from the prospective and the retrospective parts were also classified according to their composition (solid, mixed, cystic), echogenicity (hypo-, iso-, hyperechoic), margin (smooth, irregular, ill-defined) and presence of calcification (none, macro-, microcalcification). 

### Statistical Analysis

All statistical tests were performed using χ²-test and Mann–Whitney *U* test. Confidence intervals were calculated by using standard formula. Results were considered to be significant with *p* < 0.05. The statistical software used was WinStat2012.1 for Excel 2019.

## 3. Results

### 3.1. Patients

Prospective part: We examined 316 patients with newly diagnosed thyroid nodules, i.e., 632 thyroid lobes, and 441 nodules ≥ 7 mm were found (1.4 nodules per patient). In 40 nodules, as part of clinical routine, fine-needle aspiration cytology (FNAC) was performed, (9.1%) which was benign in 33 patients and unclear (Bethesda 3 or 4) in 7 patients. Five of these seven patients that were operated on their thyroid glands revealed two differentiated thyroid carcinomas. One patient with benign FNA was also operated on revealing benign histology. In addition, 25 patients without FNAC were operated on revealing four more thyroid carcinomas. Overall, 6 carcinomas in 441 nodules (1.4%, 95% CI 0.6–2.7%) were found, 2 microcarcinomas, 1 papillary thyroid carcinoma, 1 follicular thyroid carcinoma and 2 medullary thyroid carcinomas, the latter both in the same patient.

Retrospective part: From a retrospective data set with 1315 histologically and/or cytologically clarified thyroid nodules, 163 TTW nodules were retrieved and assessed with regard to the position within the thyroid gland and correlated with their risk of malignancy. In total, 62 TTW nodules were malignant; the malignancy rate was 38% (95% CI 30–46%).

### 3.2. Anatomy

In the 632 thyroid lobes examined prospectively, 197 horns were found, including 114 posterior horns and 83 posteroinferior horns. In 31 thyroid glands, there was at least one posterior horn and one posteroinferior horn. A posterior horn was located in 74 cases in the right lobe compared to 40 cases in the left lobe (*p* < 0.01) and a posteroinferior horn was located in 43 cases in the right lobe and 39 cases in the left lobe (*p* = 0.68).

### 3.3. Frequency of TTW Nodules (Part 1—Prospective Part)

**Figure 4:**
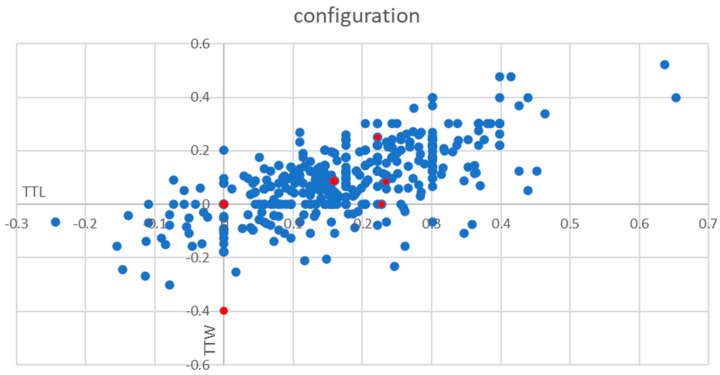
Frequency of TTW nodules. The y-axis shows the log (transversal diameter/sagittal diameter) and x-axis the log (transversal diameter/longitudinal diameter) for each nodule. The lower ends of the y-axis and the x-axis are named TTW (taller-than-wide) and TTL (taller-than-long). Red points represent malignant nodules.

Prospective part: Among a total of 441 consecutive thyroid nodules, 74 nodules were TTW (17%) and 367 nodules were non-TTW (83%) (Figure 4). In total, one of the TTW nodules was malignant (1%, CI 0–4.0%), while overall 6 out of 441 were malignant (1.4%, CI 0.6–2.7%).

### 3.4. Location of Thyroid Nodules in Thyroid Gland

TTW nodules were more often located dorsally than non-dorsally compared to non-TTW nodules (relative ratio 2.3 with a 95% CI 2.1 to 2.5; Table 1).

**Figure 5:**
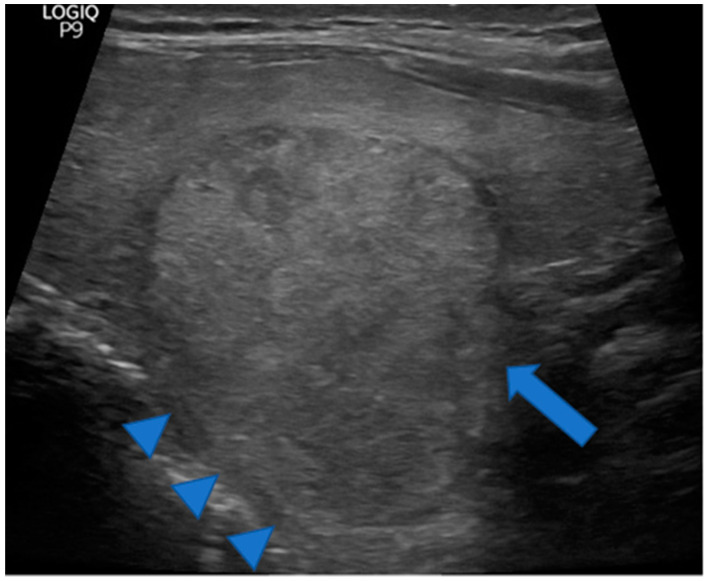
Huge TTW nodule extending into a prominent posterior horn (arrowheads). Note the thyroid parenchyma extending along the cranial portion of the nodule (arrowheads) but not along the caudal portion (arrow) arguing for a pre-existing posterior horn. A pre-existing posterior horn may have channeled the way for nodule growth causing its taller than wide shape. The nodule was benign at cytology.

Additionally, TTW nodules were more often associated with a horn than they were outside a horn compared to non-TTW nodules (relative ratio 3.0 with a 95% CI 2.4 to 3.8; Table 2, Figure 5).

**Figure 6:**
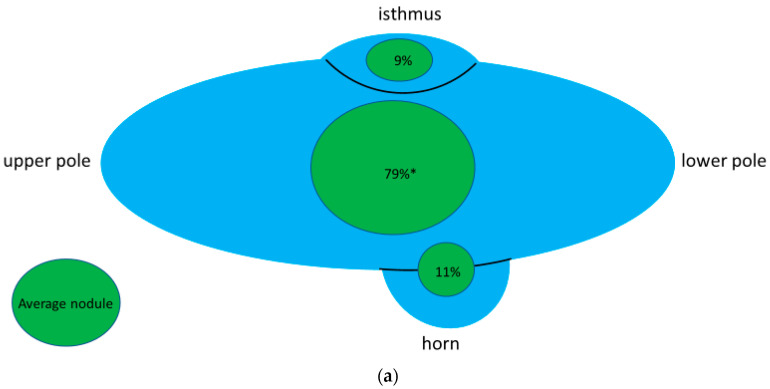

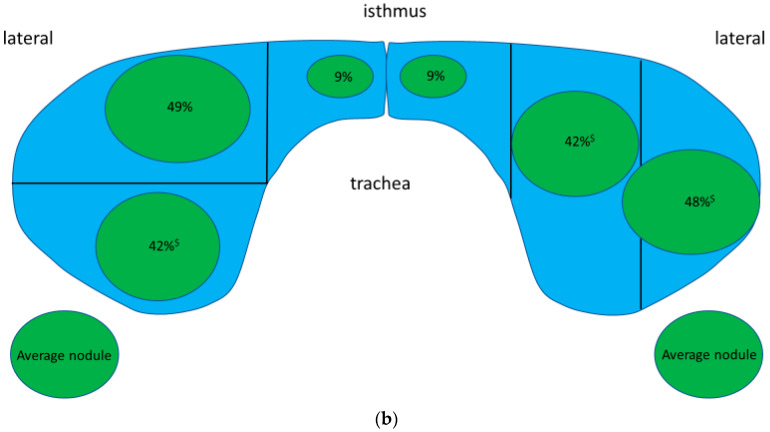
(**a**,**b**). The green circles represent typical benign nodule in the respective part of the thyroid gland. The size of each circle represents the frequency of nodules in the respective part (all parts together sum up to 100%, apart from rounding differences). The shape represents the typical relation of the sagittal diameter (tall) to the horizontal diameter (wide) of a typical nodule. Note that nodules at or in a horn are typically round (tall = wide) whereas nodules in other locations are elliptic (tall < wide) in particular in the thyroid isthmus (tall << wide). * Frequencies at the cranial, central, and caudal portions were 13%, 41%, and 25%, respectively. Configuration did not differ between these three locations for which reason they are given as one circle. ^$^ including nodules at or in a horn.

The following figure (Figure 6) gives the frequency and configuration of a typical benign nodule—excluding the six carcinomas—from the prospective part of the study with regard to its location within the thyroid gland. 

TTW nodules showed similar sonographic features to non-TTW nodules (Table 3).

### 3.5. Location and Risk of Malignancy for TTW Nodules (Part 2—Retrospective Part)

Retrospective part: From the retrospective data set, 163 TTW nodules were retrieved and assessed with regard to the position within the thyroid gland and correlated with their risk of malignancy. The results showed that a TTW nodule is 1.7 times more often benign when it is dorsal (95% CI 1.1–2.5; Table 4) and 2.5 times more often benign when associated with a horn (95% CI 1.2–5.3; Table 5). In this highly selected patient group (only histologically/cytologically clarified nodules), the overall probability of malignancy for a TTW nodule was 38% (95% CI 30–46%).

Malignant TTW nodules more often showed sonographic risk factors in terms of composition, margin, and calcification—but not for echogenicity—than benign TTW nodules (Table 6).

## 4. Discussion

In an iodine-deficient area endemic for goiter, such as Germany, in a prospective approach, TTW nodules are a frequent finding at first presentation of a patient with a rate of 17%. In such a “real-life” setting, TTW nodules were associated with a low risk of malignancy—around 1%. We observed that TTW nodules were more frequently located dorsally in the thyroid gland and more often had contact to or were found in a protrusion, which we called posterior horn or posteroinferior horn—than non-TTW nodules. Retrospectively, our data demonstrated that TTW nodules were 2.3 times more often benign when located dorsally than non-dorsally and 3.7 times more often when growing at or in a horn than without a horn. The overall probability of malignancy of TTW nodules in that retrospectively analyzed and highly preselected patient group, was as high as 39%, which is in a sharp contrast to the low number of malignant TTW nodules found in the prospective part of our study.

The presence of a horn at the back face of each lobe—either strictly posterior or posteroinferior—was noted in roughly one-third of thyroid lobes. A posterior horn was more often found on the right side than on the left side, being in good accordance with literature results on Zuckerkandl’s tubercle [24,25]. Most studies described that the incidence of Zuckerkandl’s tubercle ranges from 59 to 87% [29,30,31,32,33], but there is one report of a very low incidence of 7% [34]. Most investigators have detected Zuckerkandl’s tubercle more frequently in the right thyroid lobe [29,30,31,32,33]. Won et al., also observed Zuckerkandl’s tubercle more frequently—nearly twice as often—on the right lobe compared to the left lobe [33]. The posteroinferior form, however, is not known from previously published data. Notably, in this study, it was found as often on the right side as on left side. This may indicate a somewhat different provenance than Zuckerkandl’s tubercle and argues against the hypothesis of both forms being variants of the same origin.

On our prospective analysis, TTW nodules had a malignancy risk of 1% (CI 0–4%, see above), which does not justify FNAC based on this single US feature. However, other studies suggest malignancy probability as high as 71% for TTW nodules [5,7]. Of note, in those studies performed in countries without iodine deficiency and also in specialized centers, the a priori malignancy rate of thyroid nodules was fairly high—about 15% (Kwak et al.: 17%; Horvath et al.: 14%), which is significantly higher in magnitude than in a primary care setting. In Germany, in a primary care setting, thyroid nodule malignancy rates are as low as 0.1 to 1.0 per cent [35,36]. The striking differences in the malignancy risk of TTW nodules, therefore, can be attributed to a positive preselection in those studies, i.e., the statistical dependence of the positive predictive value from the prevalence (theorem of Bayes). The absolute malignancy risks reported for TTW nodules in those studies clearly cannot be translated into primary care. In the retrospective part of our study on histologically and/or cytologically confirmed nodules, the high overall malignancy rate for TTW nodules (39%) can also be attributed to a positive preselection. 

In the prospective part of this study, the distribution of sonographic features in terms of structure, echogenicity, margins and the presence of calcifications did not differ between TTW nodules and non-TTW nodules. This is in accordance with the above-mentioned finding that TTW is not a major risk factor in areas with a high prevalence of nodular goiter. When comparing malignant and benign TTW nodules in the retrospective part, sonographic risk factors, such as solidity, irregular margins, and microcalcifications were more frequent in malignant nodules than in benign nodules—as was to be expected. Surprisingly, this was not true for hypoechogenicity. Most likely, this observation can be attributed to a positive preselection using hypoechogenicity as a criterion for FNAC and/or thyroid surgery.

Data on the location of a thyroid nodule and a correlation with the risk of malignancy are sparce. Studies have shown that location could be an independent risk factor in predicting the risk of thyroid cancer. One study showed a significantly higher frequency of malignancy of thyroid nodules located at the upper pole (22.2%) compared to the lower pole (4.7%) and middle section of the thyroid (15.4%) [37]. Comparable to those results, Ramundo et al., reported, the upper pole location had a slightly significant association with malignancy using ACR-TIRADS (OR 6.92; 95% CI 1.02–46.90; *p* = 0.047) [38]. Duman et al., demonstrated a higher risk for malignancy in the lower and similarly upper thyroid poles [39]. Another group analyzed a total of 3241 nodules, 335 (10.3%) of which were malignant. They found a nodule location in the thyroid isthmus to carry the highest risk of cancer diagnosis and lower lobe nodules the lowest risk [40]. So far, our results—showing TTW nodules located dorsally and associated with a horn to be more often benign than at other locations—are in good accordance with these reports. However, none of these reports took the configuration of nodules into consideration. 

**Figure 7:**
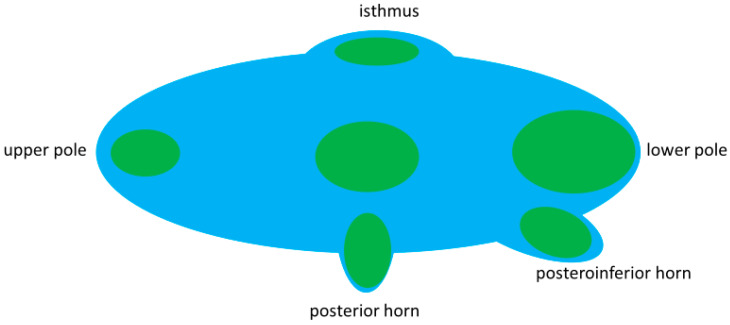
Pole concept of goiter growth. Nodule growth follows the shape of the respective pole. Overstressed depiction of nodule growth along the pole model.

The common dorsal position of benign TTW nodules and their association with a horn may modify the common assumption of benign nodules to grow parallel with the long axis of the thyroid and malignant ones to grow non-parallel. Our observation of benign nodules in the thyroid isthmus having an elliptic shape in contrast to a rounder shape at the back of the thyroid prompts a pole model for nodule growth. The model (stylized longitudinal section through a thyroid lobe, Figure 7) assumes, that the growth direction of benign nodules is predetermined by the poles of the gland. Poles in this model—besides the lower extremity, upper extremity and thyroid isthmus—are the posterior horn and the posteroinferior horn. The model assumes, that the growth of benign nodules follows the configuration of the poles. In a horn, this means a benign nodule to grow into depth rather than into width. In the center of the thyroid gland nodules follows the longitudinal oval form of the lobe. Since nodules are frequently found dorsally in the thyroid gland, i.e., near any form of a visible or non-visible horn, the pole model not only explains a TTW growth of nodules with contact to a horn but also explains the rather high frequency of TTW nodules in general. 

## 5. Conclusions

TTW nodules are common in an endemic area for goiter and appear to have a low risk for malignancy, at least on a primary care level. Often located in the posterior part of the thyroid gland, they frequently have contact to a dorsally situated posterior or posteroinferior horn. The frequency of TTW nodules and their predominant benignity may be explained by a pole concept for nodule growth.

The striking difference between the discovery of low malignancy risk of TTW nodules at a primary care level and in published studies on TIRADS can be explained by a positive preselection in the latter. On a primary care level, in the absence of other US features, TTW nodules should not a priori be considered as suspicious for malignancy, at least when seen at dorsocaudal locations or when associated with a horn.

## Figures and Tables

**Table 1:** Location of TTW nodules and non-TTW nodules in thyroid gland.

Location	TTW Nodules (*n* = 74)	Non-TTW Nodules (*n* = 367)
dorsal position (*n* = 216)	51	165
non-dorsal position (*n* = 225)	23	202

*p* = 0.01.

**Table 2:** TTW nodules and non-TTW nodules in association with a horn.

Relationship with Horn	TTW Nodules (*n* = 74)	Non-TTW Nodules (*n* = 367)
association to horn (*n* = 51)	21	30
no association to horn (*n* = 390)	53	337

*p* = 0.01.

**Table 3:** Ultrasound features in non-TTW nodules and TTW nodules.

Category		Non-TTW Nodules (*n* = 367) %	TTW Nodules (*n* = 74) %
composition	*solid*	71	75
	*mixed*	16	12
	*spongiform*	8	6
	*cystic*	6	7
echogenity	*hypoechoic*	34	32
	*isoechoic*	52	51
	*hyperechoic*	7	9
	*anechoic*	8	9
margin	*smooth*	91	81
	*lobulated/irregular/ill-defined*	9	19
calcification	*none*	72	68
	*microcalcification*	3	6
	*other*	25	26

*p* > 0.3 (for each category).

**Table 4:** Association between location and malignancy rate of TTW nodules.

Location	Malignant TTW Nodules (*n* = 62)	Benign TTW Nodules (*n* = 101)
dorsal position (*n* = 78)	22	56
non-dorsal position (*n* = 85)	40	45

*p* = 0.019.

**Table 5:** Relation between association to a horn and malignancy rate of TTW nodules.

Relationship with Horn	Malignant TTW Nodules (*n* = 62)	Benign TTW Nodules (*n* = 101)
association to horn (*n* = 35)	6	29
no association to horn (*n* = 128)	56	72

*p* = 0.003.

**Table 6:** Sonographic features in malignant and benign TTW nodules.

Category		Malignant TTW Nodules (*n* = 62) %	Benign TTW Nodules (*n* = 101) %
composition	*solid*	96	85
	*mixed*	4	14
	*cystic*	0	1
echogenicity	*hypoechoic*	85	65
	*isoechoic*	15	34
	*hyperechoic*	0	0
	*anechoic*	0	1
margin	*smooth*	22	75
	*lobulated/irregular/ill-defined*	78	25
calcification	*none*	31	62
	*microcalcification*	51	20
	*other*	18	18

*p* < 0.01, except for echogenicity (*p* = 0.39).

## Data Availability

The data that support the findings of this study are available from the corresponding author upon reasonable request.
